# The Value of Anti-Malarial Public Works

**Published:** 1906-07-14

**Authors:** 


### THE VALUE OF ANTI-MALARIAL PUBLIC WORKS.

Most Colonial medical officers are aware of the great advantages of extensive draining and other operations necessary in malarious countries in keeping down the incidence of attack both among the natives and the white residents, and the results fully justify the expenditure undertaken by Government. This is further illustrated by a report from Klang and Port Swettenham, a moist, swampy, rubber district in the Malay States. At the latter place a large area has been cleared and drained and levelled and covered with grass at a total cost of £7,000. At Klang similar measures were taken, costing £3,100. The improvement in the health of the inhabitants began immediately after the completion of the work, and has since continued. Thus at these two places in 1901 deaths from malaria amounted to 368; last year only 45; whereas in the rest of the district, which has not been dealt with by any anti-malarial works, in 1901 the deaths amounted to 266; last year, 351—a distinct increase. Formerly the bush was considered the safe place in a malarious country; now, thanks to recent research, the town dwellers are relatively in the safer position. Malaria has not died out. In the areas treated it has practically ceased to exist, but in other areas it has actually increased. This experience should be of value to those responsible for the health of communities similarly situated in many other parts of the world.

